# Quantitative acetylated proteomics on left atrial appendage tissues revealed atrial energy metabolism and contraction status in patients with valvular heart disease with atrial fibrillation

**DOI:** 10.3389/fcvm.2022.962036

**Published:** 2022-09-13

**Authors:** Tao Tu, Fen Qin, Fan Bai, Yichao Xiao, Yingxu Ma, Biao Li, Na Liu, Baojian Zhang, Chao Sun, Xiaobo Liao, Shenghua Zhou, Qiming Liu

**Affiliations:** ^1^Department of Cardiovascular Medicine, The Second Xiangya Hospital of Central South University, Changsha, China; ^2^Department of Cardiology, The First Affiliated Hospital of Zhengzhou University, Zhengzhou, China; ^3^Department of Cardiovascular Surgery, The Second Xiangya Hospital of Central South University, Changsha, China

**Keywords:** atrial fibrillation, left atrial appendage, acetylation, proteomics, energy metabolism, contraction

## Abstract

**Background:**

Numerous basic studies have demonstrated critical roles of metabolic and contractile remodeling in pathophysiological changes of atrial fibrillation (AF), but acetylation changes underlying atrial remodeling have not been fully elucidated. Quantitative acetylated proteomics enables researchers to identify a comprehensive map of protein alterations responsible for pathological development and progression of AF in the heart of patients.

**Materials and methods:**

In this study, 18 samples (9 with chronic AF and 9 with sinus rhythm) of left atrial appendage (LAA) tissues were obtained during mitral valve replacement surgery. Changes in the quantitative acetylated proteome between the AF and sinus rhythm (SR) groups were studied by dimethyl labeling, acetylation affinity enrichment, and high-performance liquid chromatography-tandem mass spectrometry analysis.

**Results:**

We identified a total of 5,007 acetylated sites on 1,330 acetylated proteins, among which 352 acetylated sites on 193 acetylated proteins were differentially expressed between the AF and SR groups by setting a quantification ratio of 1.3 for threshold value and *P* < 0.05 for significant statistical difference. The bioinformatics analysis showed that the differentially expressed acetylated proteins were mainly involved in energy metabolism and cellular contraction and structure function-related biological processes and pathways. Among 87 differentially expressed energy metabolism acetylated proteins related to the processes of fatty acid, carbohydrate, ketone body metabolism, and oxidative phosphorylation, nearly 87.1% Kac sites were upregulated (148 Kac sites among 170) in the AF group. Besides, generally declining acetylation of cardiac muscle contraction-related proteins (88.9% Kac sites of myosin) was found in the LAA of patients with AF. Immune coprecipitation combined with Western blotting was conducted to validate the differential expression of acetylated proteins.

**Conclusion:**

Many differentially expressed energy metabolism and cellular contraction acetylated proteins were found in the LAA tissues of patients with chronic AF, and may reflect the impaired ATP production capacity and decreased atrial muscle contractility in the atrium during AF. Thus, acetylation may play an important regulatory role in metabolic and contractile remodeling of the atrium during AF. Moreover, the identified new acetylated sites and proteins may become promising targets for prevention and treatment of AF.

## Introduction

Atrial fibrillation (AF), the most common arrhythmia in the clinic, is associated with increased prevalence and higher risk of thromboembolic events and heart failure, resulting in elevated morbidity and mortality (1, 2). AF is a self-perpetuating arrhythmia that is called “AF begets AF” (3), and a significant proportion of patients with paroxysmal AF develop into persistent or even permanent AF over time, but the underlying mechanisms remain unclear (4). A variety of cardiac diseases and conditions may cause atrial remodeling, including electrical, metabolic, structural, and contractile remodeling, and consequently lead to AF development (5).

At the molecular level, atrial remodeling could be defined as an assembly of the change of hundreds of proteins in atrial tissues that jointly alter cellular processes and produce characteristic remodeling (5). Impaired ATP production capacity and increased energy consumption are thought to play central roles in initiating and promoting AF (6, 7). Multiple factors have been confirmed to be involved in the ATP capacity process, especially impaired activity of complexes I and II and mitochondrial energy metabolism-related enzymes (6, 7). Proteomics techniques, as an emerging technology, have been applied to identify protein alterations responsible for the pathophysiology that mediate AF-related remodeling (8–11). Acetylation of lysine is a reversible posttranslational modification (PTM) that alters the charge of lysine residues and modifies protein structures, thereby influencing enzyme activity, DNA binding affinity, and protein stability (12, 13). For instance, more than 60% of mitochondrial proteins have been reported to have acetylation modification, and majority of acetylated proteins are enzymes for catalysis of energy metabolism (14, 15). Besides, lysine acetylation regulates the interaction of histones with DNA and the activity of non-histone proteins in many important cellular processes, including metabolism, oxidative stress, cellular contraction, signal transduction, and cell survival (16–21). Posttranslational acetylation-mediated modification is thought to regulate the energy metabolism and cellular contractility of cardiomyocytes (22, 23). Several studies have uncovered altered lysine acetylation in cardiovascular diseases, including coronary artery disease, arrhythmia, cardiac hypertrophy, and heart failure (23–25).

Until now, there has not been a comprehensive survey on the global acetylated proteome of human atrial tissues from patients with AF. Accordingly, in this study, we used the methods of dimethyl labeling, acetylation affinity enrichment, high-performance liquid chromatography-tandem mass spectrometry (HPLC-MS/MS) analysis, and database search combined with bioinformatic analysis to delineate a global profile of differential expression in acetylated proteome between left atrial appendage (LAA) tissues from patients with chronic AF and those with sinus rhythm (SR). Characteristically differentially expressed acetylated proteins, pathways, and networks could help us to explore the underlying mechanisms of AF in the context of acetylated proteomics, further providing new therapeutic targets for prevention and treatment of AF.

## Materials and methods

### Patient selection and tissues samples

This study included 9 cases of patients with mitral valvular heart disease with chronic AF (documented persistent AF for at least 1 year and permanent AF, the AF group) and with SR (the SR group, without a history of AF) who undergone mitral valve replacement surgery at The Second Xiangya Hospital of Central South University. The SR group patients were screened to ensure that they had never experienced AF by direct questioning about symptoms suggestive of AF and 12-lead electrocardiography during the preoperative review period. Informed consent was obtained from all the patients recruited in this study for their LAA tissue samples to be studied. Patients with diabetes mellitus and hyperlipemia were excluded from this study. All the patients received routine transthoracic ultrasonography before surgery, and patients aged >50 years routinely undergone a coronary angiography, by which those with coronary artery disease were excluded. The clinical baseline characteristics of the 2 groups were collected to be statistically analyzed. The LAA tissue samples were harvested and obtained after ligation of LAA while the patients were undergoing mitral valve replacement surgery and then immediately snap-frozen and stored in liquid nitrogen.

### Protein extraction

This study mixed three LAA tissues from the AF group or the SR group as one sample to obtain 3 samples for the AF group or the SR group. Paired comparison between the 2 groups was done to get AF-1/SR-1, AF-2/SR-2, and AF-3/SR-3 comparisons. Each sample was ground into powder with liquid nitrogen and then transferred to a 5-ml centrifuge tube. After that, four volumes of a lysis buffer (8 M urea, 1% protease inhibitor cocktail, 3 μM trichostatin A, 50 mM nicotinamide, and 2 mM ethylene diamine tetraacetic acid) was added to the powder, followed by sonication thrice on ice using a high-intensity ultrasonic processor (Scientz). The remaining debris was removed by centrifugation at 12,000 *g* at 4°C for 10 min. Finally, the supernatant was collected, and the protein concentration was determined with a BCA kit according to the manufacturer’s instructions.

### Trypsin digestion

For trypsin digestion, the protein solution was reduced with 5 mM dithiothreitol for 30 min at 56°C and alkylated with 11 mM iodoacetamide for 15 min at room temperature in darkness. The protein sample was then diluted by adding 100 mM TEAB to urea concentration less than 2 M. Finally, trypsin was added at 1:50 trypsin-to-protein mass ratio for the first digestion overnight and 1:100 trypsin-to-protein mass ratio for the second 4-h digestion.

### Dimethyl labeling

After trypsin digestion, peptides were desalted with a Strata X C18 SPE column (Phenomenex) and vacuum-dried. Peptides from the AF group and the SR group were dissolved in 0.5 M triethylammonium bicarbonate solution and separately labeled as heavy or light dimethyl group according to the manufacturer’s protocol for the dimethyl labeling kit (Thermo Fisher Scientific, United States). Briefly, the labeled reagent was thawed and dissolved in acetonitrile and the peptide samples were differentially isotope dimethyl labeled in parallel in different tubes, about 2 mg of each sample was used for labeling. 320 μL of 4% CD2O for AF group or CH2O for SR group was added to the peptides to be labeled with heavy and light dimethyl, respectively. The peptides were incubated with the labeling reagent for 2 h at room temperature, and then the labeled peptides were desalted and vacuum-dried again.

### High-performance liquid chromatography fractionation

Tryptic peptides were fractionated by high pH reverse-phase HPLC using a ThermoBetasil C18 column (5 μm particles, 10 mm ID, 250 mm length). Briefly, the peptides were first separated with a gradient of 8 to 32% acetonitrile (pH 9) over 60 min into 60 fractions. Then, the peptides were combined into 4 fractions and dried by vacuum centrifugation.

### Affinity enrichment

To enrich lysine acetylated (Kac) peptides, the tryptic peptides were dissolved in a NETN buffer (100 mM NaCl, 1 mM EDTA, 50 mM Tris-HCl, and 5% NP-40, pH 8) and incubated with pre-washed anti-acetyl lysine antibody beads (PTM Biolabs, China) overnight with gentle shaking at 4°C. Then, the peptide-enriched beads were washed four times with the NETN buffer and twice with ddH_2_O. Next, the bound peptides were eluted from the beads with 0.1% trifluoroacetic acid. Finally, the eluted fractions were combined and vacuum-dried, and the resulting peptides were desalted with C18 ZipTips (Millipore) according to the manufacturer’s instructions for further-high performance liquid chromatography-tandem mass spectrometry (HPLC-MS/MS) analysis.

### High-performance liquid chromatography-tandem mass spectrometry analysis

The tryptic peptides were dissolved in 0.1% formic acid (solvent A) and directly loaded onto a homemade reversed-phase analytical column (15 cm length, 75 μmi.d.). The gradient was comprised of an increase from 6 to 23% solvent B (0.1% formic acid in 98% acetonitrile) over 26 min, 23 to 35% in 8 min and climbing to 80% in 3 min then holding at 80% for the last 3 min, all at a constant flow rate of 400 nl/min on an EASY-nLC 1000 UPLC system.

The peptides were subjected to NSI source followed by a tandem mass spectrometry (MS/MS) in Q Exactive™ Plus (Thermo) coupled online to UPLC. The electrospray voltage applied was 2 kV. The m/z scan range was 350 to 1,800 for full scan, and intact peptides were detected in the Orbitrap at a resolution of 70,000. Peptides were then selected for MS/MS using an NCE setting of 28, and fragments were detected in the Orbitrap at a resolution of 17,500. A data-dependent procedure that alternated between one MS scan followed by 20 MS/MS scans with 15.0 s dynamic exclusion. Automatic gain control (AGC) was set at 5E4. Fixed first mass was set at 100 m/z.

### Database search

The acquired MS/MS data were processed using Maxquant with the integrated Andromeda search engine (v.1.5.2.8). Tandem mass spectra were searched against the SwissProt Human database (20,317 sequences) concatenated with a reverse decoy database. Trypsin/P was specified as cleavage enzyme allowing up to 4 missing cleavages. The minimum length of the peptides was set at 7 amino acid residues, and the maximum modification number of the peptides was set at 5. The mass tolerance for precursor ions was set at 20 ppm in the first search and 5 ppm in the main search, and the mass tolerance for fragment ions was set at 0.02 Da. Carbamidomethylation on Cys was accounted for as fixed modification, and oxidation on Met, acetylation on lysine, and acetylation on protein N-terminal were accounted for as variable modifications. False discovery rate (FDR) was adjusted to <1%, and minimum score for modified peptides was set >40.

### Bioinformatics analysis

Gene ontology (GO) annotation was derived from the UniProt-GOA database^1^. If some identified proteins were not annotated by the UniProt-GOA database, the InterProScan software (v.5.14–53.0^2^) was used to annotate a protein’s GO function based on protein sequence alignment method. Proteins were classified by GO annotation based on three categories: molecular function, cellular component, and biological process. The Wolfpsort software (v.0.2^3^), an updated version of PSORT/PSORT II for prediction of eukaryotic sequences, was used to predict subcellular localization. The Kyoto Encyclopedia of Genes and Genomes (KEGG) database^4^ was used to annotate protein pathways. First, the KEGG online service tool KAAS (v.2.0^5^) was used to annotate a protein’s KEGG database description. Then, the KEGG online service tool KEGG mapper (v.5^6^) was used to map the annotation results. Protein domain annotation was derived from the InterPro domain database (v.5.14–53.0, see text footnote 2). The manually curated human CORUM protein complex database (v.2.0^7^) was selected to analyze protein complexes.

The enrichment analysis was determined by two-tailed Fisher’s exact test using the Perl module (v.1.31^8^) to identify the enrichment of the differentially expressed proteins against the background of all identified proteins with a corrected *P*-value < 0.05. An enrichment-based clustering analysis was conducted according to protein functional classification (GO, KEGG pathway, protein domain, and protein complex). We first collated all the categories obtained after enrichment along with their *P*-values and then filtered for categories that were at least enriched in one of the clusters with *P*-value < 0.05. The filtered *P*-value matrix was transformed with the function *x* = –log10 (*P*-value). Finally, the *x*-values were z-transformed for each functional category. The z scores were then clustered by one-way hierarchical clustering (Euclidean distance, average linkage clustering) in Genesis. Cluster membership was visualized with a heat map using the “heatmap.2” function from the “gplots” R-package (v.2.0.3^9^).

All differentially expressed protein sequences were searched against the search tool for the Retrieval of Interacting Genes/Proteins (STRING) database (v.10.5) for protein–protein interactions. Only interactions between proteins belonging to the searched data set were selected, thereby excluding external candidates. STRING defines a metric called “confidence score” to describe interaction confidence, and we fetched all interactions that had a confidence score ≥0.7 (high confidence). The interaction network form STRING was visualized in the network analysis and with visualization software “R package networkD3” (v.0.4^10^). A graph clustering algorithm, molecular complex detection (MCODE), was utilized to analyze densely connected regions. Soft MoMo (v.5.0.2^11^) was used to analyze the model of sequences constituting amino acids in specific positions of acetyl-21-mers (10 amino acids upstream and downstream of the Kac site) in all identified Kac protein sequences.

### Co-immunoprecipitation and western blotting analysis

We conducted co-immunoprecipitation (Co-IP) combined with Western blotting (WB) to validate the differential expression of acetylated proteins in the LAA tissue samples from the patients with AF and SR. Co-IP was performed by following the Pierce Classic IP kit (Thermo Fisher Scientific, United States) protocol. The anti-acetyl lysine rabbit pAb was custom-prepared (PTM Biolabs, China). Fifty μg of protein was separated by 10% sodium dodecyl sulfate-polyacrylamide gel electrophoresis and transferred to Hybond-P polyvinylidene difluoride membranes (Amersham Biosciences, Uppsala, Sweden). After blocking with 5% non-fat dry milk in Tris-buffered saline buffer for 1 h at room temperature, the blots were incubated at room temperature with 1:800 diluted primary antibodies (mouse antibody) (Abcam, Cambridge, MA, United States) followed by appropriate secondary antibodies (goat-anti-mouse antibody) (Abcam, Cambridge, MA, United States) for 1 h. Signals were visualized with an enhanced chemiluminescence detection reagent (Abcam).

## Results

### Patients’ clinical baseline characteristics

The clinical baseline characteristics of the AF and SR group patients are presented in Table 1. There were no significant differences in clinical baseline characteristics between the 2 groups of patients (*P* > 0.05), except the left atrium diameter was significantly larger in the AF group than in the SR group (49.4 ± 8 mm vs. 37.9 ± 3.1 mm, *p* = 0.001).

**TABLE 1:** Clinical baseline characteristics of the AF and SR group patients.

	AF group (*N* = 9)	SR group (*N* = 9)	*p*
Male (*N*, %)	4 (44.4%)	5 (55.6%)	1.000
Age (years)	55.5 ± 9.0	50.5 ± 6.5	0.195
BMI (kg/m^2^)	22.7 ± 1.8	22.2 ± 2.0	0.489
Hypertension (*N*, %)	6 (66.7%)	4 (44.4%)	0.637
NT-proBNP (pg/mL)	608.4 ± 687.9	689.2 ± 653.1	0.605
Fasting blood glucose (mmol/L)	5.2 ± 0.4	5.0 ± 0.4	0.489
Total cholesterol (mmol/L)	4.3 ± 0.5	4.6 ± 0.5	0.269
Mitral valve area (cm^2^)	1.9 ± 0.3	1.8 ± 0.3	0.746
RAD (mm)	33.4 ± 5.3	33.0 ± 4.3	0.796
LAD (mm)	49.4 ± 8.0	37.9 ± 3.1	0.001*
RVEDD (mm)	33.8 ± 8.1	30.1 ± 4.9	0.489
LVEDD (mm)	54.1 ± 10.8	46.9 ± 10.6	0.161
LVEF (%)	61.3 ± 8.8	62.9 ± 8.6	0.711

AF, atrial fibrillation; SR, sinus rhythm; BMI, body mass index; NT-proBNP, N-terminal pro brain natriuretic peptide; RAD, right atrial dimension; LAD, left atrial dimension; RVEDD, right ventricular end-diastolic dimension; LVEDD, left ventricular end-diastolic dimension; LVEF, left ventricular ejection fraction. *P < 0.05.

### Quality testing

**FIGURE 1:**
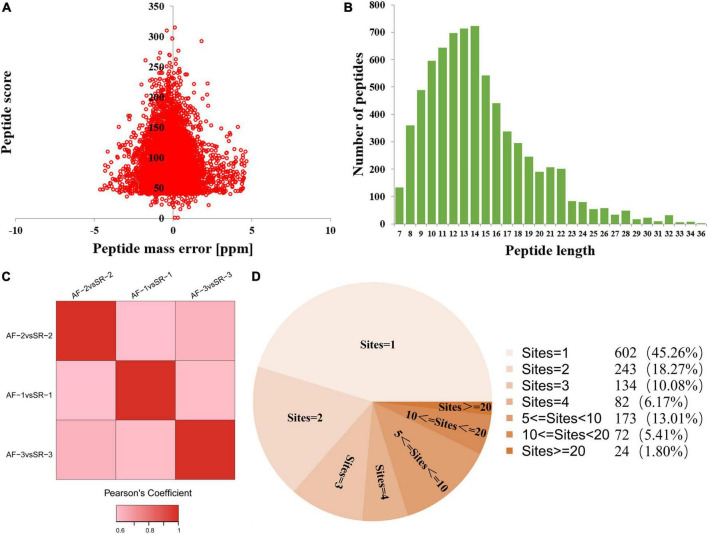
Quality testing and identification of acetylated proteome in LAA tissues. **(A)** Mass error distribution of all identified peptides. **(B)** Peptide length distribution of all identified peptides. **(C)** Repeatability testing. **(D)** Pie chart showing the distribution of the number of Kac site identifications per Kac protein. AF, atrial fibrillation; SR, sinus rhythm; LAA, left atrial appendage.

The quality testing of all identified Kac peptides found that the distribution of mass errors of Kac peptides was near zero, and that most were ≤5 ppm (Figure 1A), suggesting that the mass accuracy of the MS data fits the requirement. Moreover, all Kac peptides were between 8 and 32 amino acids in length, which was consistent with tryptic peptides (Figure 1B). These results indicated that the sample preparation method was adequate, and that the modified peptide data obtained from MS were highly accurate. Pearson’s correlation analysis for repeatability testing indicated that the 3 AF/SR comparisons had good repeatability (Figure 1C).

### Identification and atrial fibrillation/sinus rhythm differentially expressed analysis of acetylated proteome

This study identified a total of 5,007 Kac sites on 1,330 Kac proteins, among which 3,880 sites on 1,044 proteins were quantified. A total of 602 Kac proteins (45.26%) had only 1 Kac site, and the rest of 728 Kac proteins had more than 1 Kac site. The distribution of the number of Kac site identifications per Kac protein is shown in Figure 1D. The first 3 Kac proteins with most numbers of Kac sites were titin (TTN) with 407 sites, myosin-6 (MYH6) with 105 sites, and myosin-7 (MYH7) with 103 sites.

**FIGURE 2:**
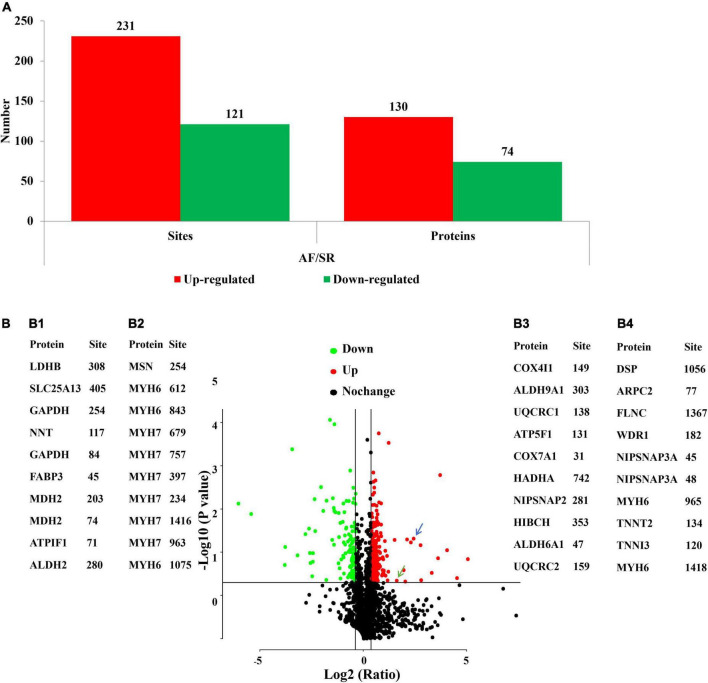
AF/SR differentially expressed analysis of acetylated proteome in LAA tissues. **(A)** Bar graph showing the number of upregulated (AF/SR ratio >1.3) and downregulated (AF/SR ratio <1/1.3) Kac sites and Kac proteins. **(B)** Volcano plots of the *p-*values vs. the log_2_ Kac protein abundance differences between LAA tissues from the AF and SR group patients, with Kac proteins outside the significance line colored in red (upregulated) or green (downregulated) (false discovery rate <0.05). **(B1,B2)** show the top 10 downregulated Kac sites on Kac proteins related to energy metabolism and cellular contraction, respectively; **(B3,B4)** show the top 10 upregulated Kac sites on Kac protein related to energy metabolism and cellular contraction, respectively. The blue arrow represents cytochrome c oxidase subunit 5A, mitochondrial (COX5A) in energy metabolism-related proteins, and the green arrow represents vinculin (VCL) in cellular contraction-related proteins. *P*-values were calculated from the data of the acetylated proteome in LAA tissues from the AF and SR group patients. AF, atrial fibrillation; SR, sinus rhythm; LAA, left atrial appendage.

The acetylated levels of 352 Kac sites on 193 Kac proteins were differentially expressed between the AF group and the SR group by setting a quantification ratio of 1.3 for threshold value and *P* < 0.05 for significant statistical difference. Furthermore, 231 upregulated (AF/SR ratio >1.3) Kac sites on 130 Kac proteins and 121 downregulated (AF/SR ratio <1/1.3) Kac sites on 74 Kac proteins were detected in the AF group (Figures 2A,B). Relevant information on top 10 upregulated and downregulated Kac sites on Kac proteins in the AF group is shown in Table 2. Furthermore, in Figures 2B,B1,B2 show the top 10 downregulated Kac sites on Kac proteins related to energy metabolism and cellular contraction, respectively; B3 and B4 show the top 10 upregulated Kac sites on Kac proteins related to energy metabolism and cellular contraction, respectively.

**TABLE 2:** Top 10 upregulated and downregulated Kac sites in Kac proteins in the AF group.

Regulated Type	Rank	Gene	Protein accession	Protein description	Position	AF/SR Ratio	AF/SR *P*-value
Up	1	GFM2	Q969S9	“Ribosome-releasing factor 2, mitochondrial”	233	32.893	0.0146
Up	2	LMNA	P02545	Prelamin-A/C	32	22.887	0.04
Up	3	GMPR	P36959	GMP reductase 1	13	16.383	0.00906
Up	4	COX4I1	P13073	“Cytochrome c oxidase subunit 4 isoform 1, mitochondrial”	149	12.999	0.000166
Up	5	ALDH9A1	P49189	4-trimethylaminobutyraldehyde dehydrogenase	303	12.107	0.0139
Up	6	UQCRC1	P31930	“Cytochrome b-c1 complex subunit 1, mitochondrial”	138	9.809	0.0303
Up	7	ATP5F1	P24539	“ATP synthase F(0) complex subunit B1, mitochondrial”	131	6.872	0.0448
Up	8	DSP	P15924	Desmoplakin	1056	6.739	0.00694
Up	9	ARPC2	O15144	Actin-related protein 2/3 complex subunit 2	77	5.337	0.0049
Up	10	COX7A1	P24310	“Cytochrome c oxidase subunit 7A1, mitochondrial”	31	4.869	0.00598
Down	1	MSN	P26038	Moesin	254	0.015	0.000758
Down	2	COL6A1	P12109	Collagen alpha-1 (VI) chain	61	0.023	0.00132
Down	3	LDHB	P07195	L-lactate dehydrogenase B chain	308	0.071	0.0199
Down	4	MYH6	P13533	Myosin-6	612	0.072	0.00763
Down	5	MYH6	P13533	Myosin-6	843	0.091	0.000042
Down	6	SLC25A13	Q9UJS0	Calcium-binding mitochondrial carrier protein Aralar2	405	0.109	0.0119
Down	7	IGHM	P01871	Immunoglobulin heavy constant mu	131	0.142	0.00385
Down	8	PRDX1	Q06830	Peroxiredoxin-1	168	0.16	0.00287
Down	9	GAPDH	P04406	Glyceraldehyde-3-phosphate dehydrogenase	254	0.165	0.0105
Down	10	NNT	Q13423	“NAD(P) transhydrogenase, mitochondrial”	117	0.168	0.0179

AF, atrial fibrillation; SR, sinus rhythm.

### Annotation classification of gene ontology and subcellular location of the atrial fibrillation/sinus rhythm differentially expressed acetylated proteome

**FIGURE 3:**
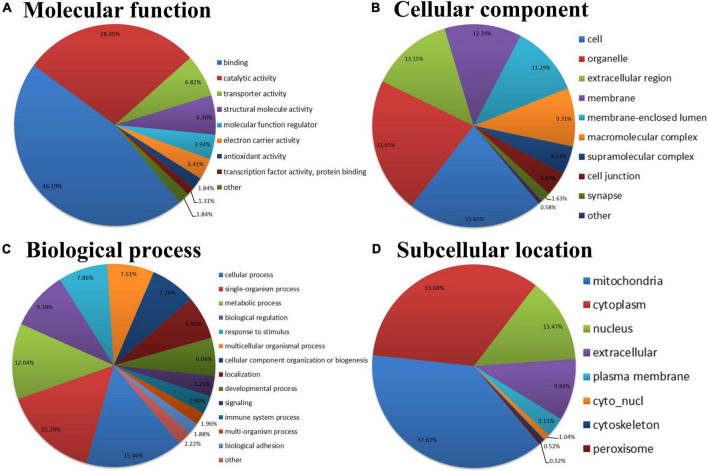
GO and subcellular location annotation classification of the AF/SR differentially expressed acetylated proteome in LAA tissues. **(A)** Molecular function. **(B)** Cellular component. **(C)** Biological process. **(D)** Subcellular location. GO, gene ontology; AF, atrial fibrillation; SR, sinus rhythm; LAA, left atrial appendage.

To better understand the biological feature of AF/SR differentially expressed Kac proteins, GO classification and subcellular location analysis were performed (Figure 3). In the molecular function analysis, we found that proteins related to binding (46.19%), catalytic activity (28.35%), and transporter activity (6.82%) were predominantly enriched (Figure 3A). The 3 principal cellular components were cell (21.65%), organelle (21.65%), and extracellular region (13.15%) (Figure 3B). In the biological process category, proteins were highly enriched in the cellular process (15.46%), followed by single organism process (15.29%), and metabolic process (12.04%) (Figure 3C). The subcellular location classification indicated that the differentially expressed Kac proteins were predominantly located in the mitochondria, cytoplasm, and nucleus (Figure 3D).

### Enrichment analysis of gene ontology, Kyoto Encyclopedia of Genes and Genomes pathway, protein domain, and protein complex of the atrial fibrillation/sinus rhythm differentially expressed acetylated proteome

**FIGURE 4:**
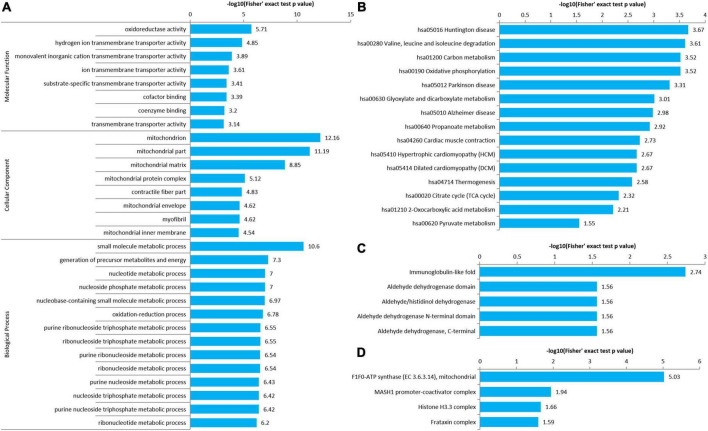
Enrichment analysis of GO, KEGG pathway, protein domain, and protein complex of the AF/SR differentially expressed acetylated proteome in LAA tissues. **(A)** GO (molecular function, cellular component, and biological process). **(B)** KEGG pathway. **(C)** Protein domain. **(D)** Protein complex. GO, gene ontology; KEGG, Kyoto Encyclopedia of Genes and Genomes; AF, atrial fibrillation; SR, sinus rhythm; LAA, left atrial appendage.

An enrichment analysis of GO, KEGG pathway, protein domain, and protein complex was performed to identify the enrichment of AF/SR differentially expressed Kac proteins including upregulated (AF/SR ratio >1.3) and downregulated (AF/SR ratio <1/1.3) proteins against the background of all identified proteins (Figure 4).

The molecular function, cellular component category, and biological process classification enrichment analysis of the AF/SR differentially expressed proteins is shown in Figure 4A. Enriched Kac proteins are prominently distributed in mitochondria and mainly involved in oxidoreductase activity, transporter activity, and multiple key metabolic processes like energy and nucleoside phosphate metabolic processes (Figure 4A).

The KEGG pathway enrichment analysis of AF/SR differentially expressed Kac proteins showed main related pathways, such as carbon metabolism, oxidative phosphorylation, and cardiac muscle contraction (Figure 4B).

In addition, a further analysis was performed for upregulated Kac proteins (Supplementary Figure 1) and downregulated Kac proteins (Supplementary Figure 2), separately.

### Enrichment-based clustering analysis of gene ontology, Kyoto Encyclopedia of Genes and Genomes pathway, protein domain, and protein complex of the atrial fibrillation/sinus rhythm differentially expressed acetylated proteome

**FIGURE 5:**
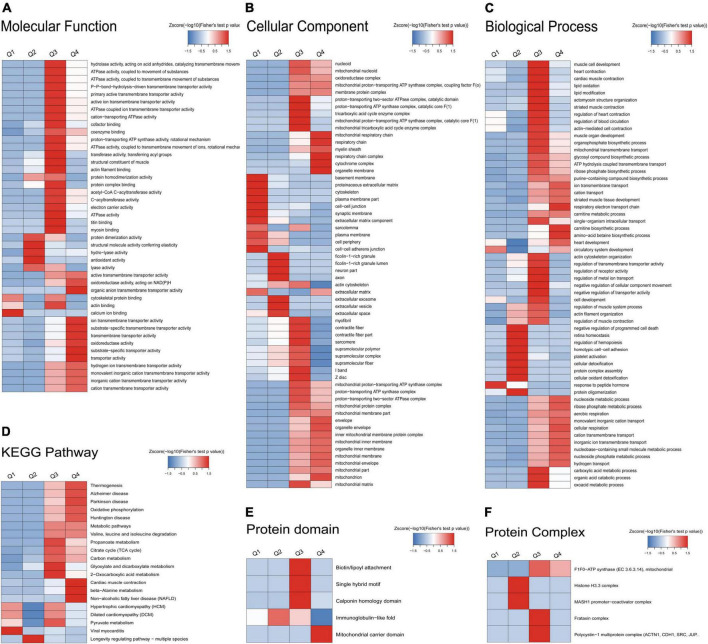
Enrichment-based clustering analysis of GO, KEGG pathway, protein domain, and protein complex of the AF/SR differentially expressed acetylated proteome in LAA tissues. **(A)** Molecular function. **(B)** Cellular component. **(C)** Biological process. **(D)** KEGG pathway. **(E)** Protein domain. **(F)** Protein complex. GO, gene ontology; KEGG, Kyoto Encyclopedia of Genes and Genomes; AF, atrial fibrillation; SR, sinus rhythm; LAA, left atrial appendage.

The AF/SR differentially expressed Kac proteins were divided into four categories according to AF/SR ratio (Q1: ratio <1/1.5, Q2: 1/1.5 < ratio < 1/1.3, Q3: 1.3 < ratio < 1.5, and Q4: >1.5). There were 64, 57, 134, and 97 Kac proteins in the Q1, Q2, Q3, and Q4 categories, respectively. An enrichment-based clustering analysis of GO, KEGG pathway, protein domain, and protein complex was performed (Figure 5).

In the molecular function analysis (Figure 5A), the AF/SR differentially expressed Kac proteins clustering in Q1 were involved in calcium ion binding. The AF/SR differentially expressed Kac proteins clustering in Q2 were involved in protein dimerization activity, structural molecule activity conferring elasticity, hydro-lyase activity, antioxidant activity, and lyase activity. The AF/SR differentially expressed Kac proteins clustering in Q3 were involved in hydrolase activity, ATPase activity, P-P-bond-hydrolysis-driven transmembrane transporter activity, primary active transmembrane transporter activity, active ion transmembrane transporter activity, cation-transporting ATPase activity, cofactor binding, coenzyme binding, proton-transporting ATP synthase activity, rotational mechanism, ATPase activity, coupled to transmembrane movement of ions, rotational mechanism, transferase activity, transferring acyl groups, structural constituent of muscle, actin filament binding, protein homodimerization activity, protein complex binding, acetyl-CoA C-acyltransferase activity, C-acyltransferase activity, electron carrier activity, ATPase activity, titin binding, myosin binding, and actin binding. The AF/SR differentially expressed Kac proteins clustering in Q4 were involved in active transmembrane transporter activity, organic anion transmembrane transporter activity, oxidoreductase activity, acting on NAD(P)H, ion transmembrane transporter activity, substrate-specific transmembrane transporter activity, transmembrane transporter activity, oxidoreductase activity, substrate-specific transporter activity, and transporter activity.

In the cellular component category (Figure 5B), the AF/SR differentially expressed Kac proteins clustering in Q1 were involved in basement membrane, proteinaceous extracellular matrix, cytoskeleton, plasma membrane part, cell-cell junction, synaptic membrane, extracellular matrix component, sarcolemma, plasma membrane, cell periphery, cell–cell adherens junction, and extracellular matrix. The AF/SR differentially expressed Kac proteins clustering in Q2 were involved in ficolin-1-rich granule, ficolin-1-rich granule lumen, neuron part, axon, extracellular space, extracellular vesicle, and extracellular exosom. The AF/SR differentially expressed Kac proteins clustering in Q3 were involved in proton-transporting two-sector ATPase complex, catalytic domain, proton-transporting ATP synthase complex, catalytic core F(1), tricarboxylic acid cycle enzyme complex, mitochondrial proton-transporting ATP synthase complex, catalytic core F(1), mitochondrial tricarboxylic acid cycle enzyme complex, myofibril, contractile fiber, contractile fiber part, sarcomere, supramolecular polymer, supramolecular complex, supramolecular fiber, I band, Z disk, mitochondrial proton-transporting ATP synthase complex, proton-transporting ATP synthase complex, proton-transporting two-sector ATPase complex, mitochondrial protein complex, mitochondrial membrane part, and mitochondrial matrix. The AF/SR differentially expressed Kac proteins clustering in Q4 were involved in mitochondrial respiratory chain, respiratory chain, myelin sheath, respiratory chain complex, cytochrome complex, organelle membrane, envelope, organelle envelope, inner mitochondrial membrane protein complex, mitochondrial inner membrane, organelle inner membrane, mitochondrial membrane, mitochondrial envelope, mitochondrial part, and mitochondria.

In the biological process classification (Figure 5C), the AF/SR differentially expressed Kac proteins clustering in Q4 were involved in response to peptide hormone. The AF/SR differentially expressed Kac proteins clustering in Q2 were involved in negative regulation of programmed cell death, retina homeostasis, regulation of hemopoiesis, homotypic cell-cell adhesion, platelet activation, cellular detoxification, protein complex assembly, cellular oxidant detoxification, and protein oligomerization. The AF/SR differentially expressed Kac proteins clustering in Q3 were involved in muscle cell development, heart contraction, cardiac muscle contraction, lipid oxidation, lipid modification, actomyosin structure organization, striated muscle contraction, regulation of heart contraction, regulation of blood circulation, actin-mediated cell contraction, actin cytoskeleton organization, regulation of transmembrane transporter activity, regulation of receptor activity, regulation of metal ion transport, negative regulation of cellular component movement, negative regulation of transporter activity, cell development, regulation of muscle system process, actin filament organization, regulation of muscle contraction, carboxylic acid metabolic process, organic acid catabolic process, and oxoacid metabolic process. The AF/SR differentially expressed Kac proteins clustering in Q4 were involved in ion transmembrane transport, cation transport, striated muscle tissue development, respiratory electron transport chain, carnitine biosynthetic process, amino acid betaine biosynthetic process, heart development, nucleoside metabolic process, ribose phosphate metabolic process, aerobic respiration, monovalent inorganic cation transport, cellular respiration, cation transmembrane transport, inorganic ion transmembrane transport, nucleobase-containing small molecule metabolic process, nucleoside phosphate metabolic process, and hydrogen transport.

In the KEGG pathway analysis (Figure 5D), the AF/SR differentially expressed Kac proteins clustering in Q1 were involved in viral myocarditis. The AF/SR differentially expressed Kac proteins clustering in Q2 were involved in longevity regulating pathway-multiple species. The AF/SR differentially expressed Kac proteins clustering in Q3 were involved in propanoate metabolism, citrate cycle (TCA cycle), carbon metabolism, glyoxylate and dicarboxylate metabolism, 2-oxocarboxylic acid metabolism, and pyruvate metabolism. The AF/SR differentially expressed Kac proteins clustering in Q4 were involved in thermogenesis, Alzheimer’s disease, Parkinson’s disease, oxidative phosphorylation, Huntington disease, metabolic pathways, valine, leucine, and isoleucine degradation, cardiac muscle contraction, beta-alanine metabolism, and non-alcoholic fatty liver disease (NAFLD).

In the protein domain category (Figure 5E), the AF/SR differentially expressed Kac proteins clustering in Q2 were involved in immunoglobulin-like fold. The AF/SR differentially expressed Kac proteins clustering in Q3 were involved in biotin/lipoyl attachment, single hybrid motif, and calponin homology domain. The AF/SR differentially expressed Kac proteins clustering in Q4 were involved in mitochondrial carrier domain.

In the protein complex classification (Figure 5F), the AF/SR differentially expressed Kac proteins clustering in Q2 were involved in histone H3.3 complex and MASH1 promoter-coactivator complex. The AF/SR differentially expressed Kac proteins clustering in Q3 were involved in F1F0-ATP synthase (EC 3.6.3.14), mitochondria, frataxin complex, and polycystin-1 multiprotein complex.

### Protein–protein interaction networks of the atrial fibrillation/sinus rhythm differentially expressed acetylated proteome

**FIGURE 6:**
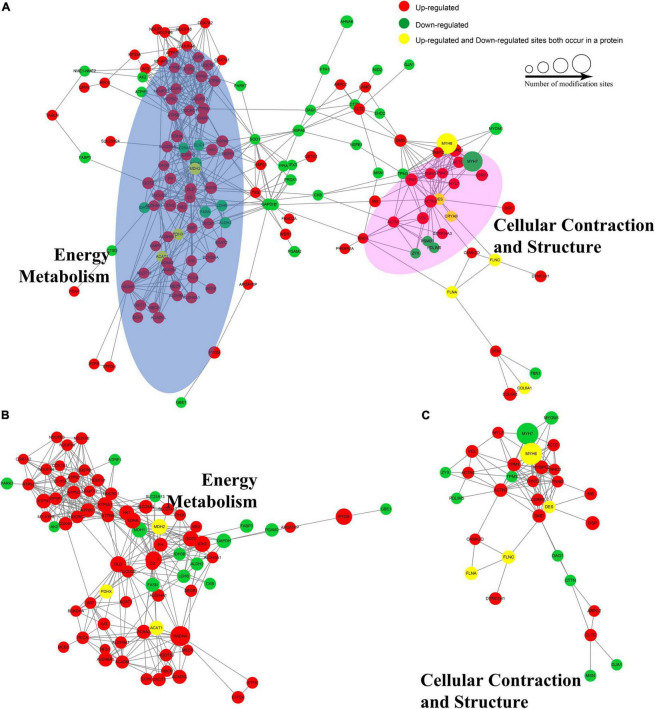
Protein–protein interaction networks of the AF/SR differentially expressed acetylated proteome in LAA tissues. **(A)** Protein–protein interaction networks of the total AF/SR differentially expressed Kac proteins. The blue oval area in panel **(A)** represents the position occupied by most of the Kac proteins related to energy metabolism in the interaction networks, and the pink area represents the cellular contraction- and structure-related Kac proteins. **(B)** Protein–protein interaction networks of the AF/SR differentially expressed Kac proteins related to energy metabolism. **(C)** Protein–protein interaction networks of the AF/SR differentially expressed Kac proteins related to cellular contraction and structure. AF, atrial fibrillation; SR, sinus rhythm; LAA, left atrial appendage.

To identify chief nodes, important connectors, and functional interactions among the AF/SR differentially expressed Kac proteins, we conducted a protein–protein interaction network analysis. There was a total of 146 nodes and 569 interactions in the protein–protein interaction network (Figure 6A). Further analysis showed that there were tight communications among Kac proteins related to energy metabolism (77 nodes and 351 interactions, Figure 6B) and among Kac proteins related to cellular contraction and structure (31 nodes and 99 interactions, Figure 6C).

### Comparison of the atrial fibrillation/sinus rhythm differentially expressed energy metabolism-related and cellular contraction and structure-related acetylated proteins and sites

**FIGURE 7:**
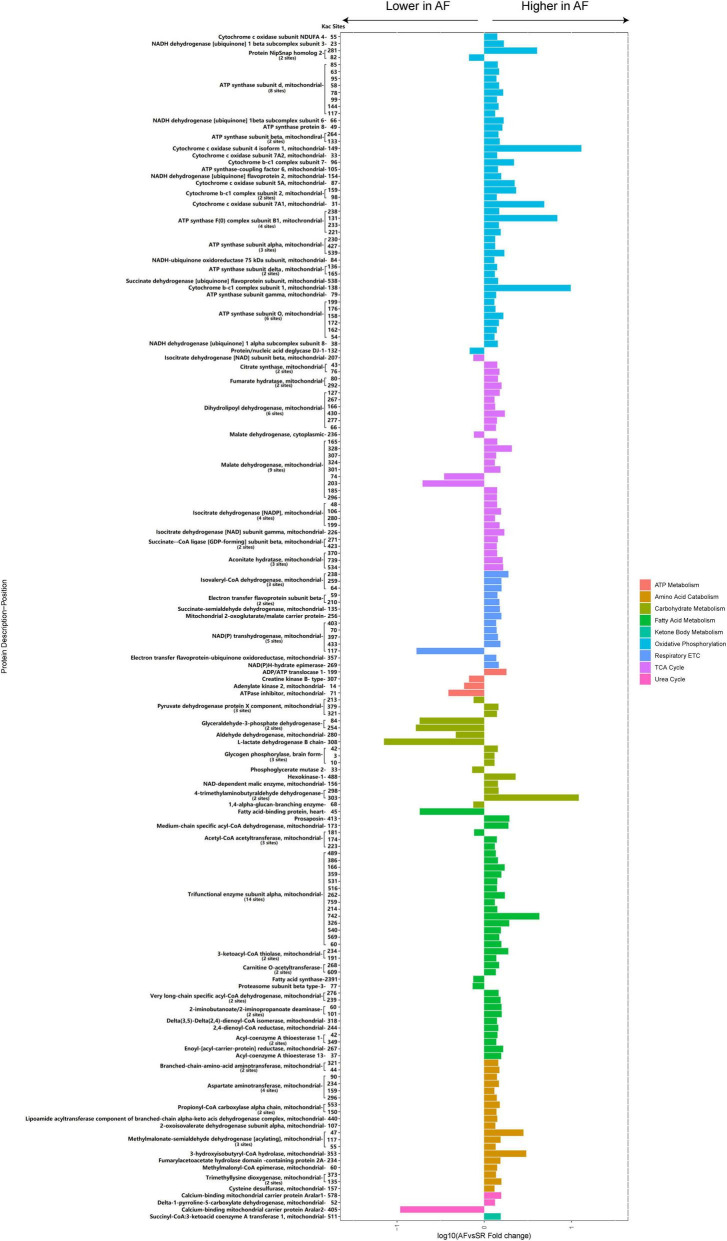
Histogram bar and comparison of the AF/SR differentially expressed energy metabolism-related acetylated proteins and sites in LAA tissues. AF, atrial fibrillation; SR, sinus rhythm; LAA, left atrial appendage; ETC, electron transport chain; TCA cycle, citrate cycle.

A total of 87 energy metabolism-related Kac proteins (accounting for 45.1% of the total AF/SR differentially expressed Kac proteins) had 170 AF/SR differentially expressed acetylated sites (accounting for 48.3% of the total AF/SR differentially expressed Kac sites) (Figure 7 and Supplementary material 1). These energy metabolism-related Kac proteins were involved in the processes of oxidative phosphorylation (46 Kac sites on 25 Kac proteins and 44 upregulated Kac sites [95.7%]), TCA cycle (31 Kac sites on 10 Kac proteins, and 27 upregulated Kac sites [87.1%]), respiratory electron transport chain (14 Kac sites on 7 Kac proteins and 13 upregulated Kac sites [92.9%]), ATP metabolism (4 Kac sites on 4 Kac proteins and 3 downregulated Kac sites [25%]), carbohydrate metabolism (16 kac sites on 10 Kac proteins and 9 upregulated Kac sites [56.3%]), fatty acid metabolism (36 Kac sites on 16 Kac proteins and 32 upregulated Kac sites [88.9%]), amino acid catabolism (19 Kac sites on 11 Kac proteins and 19 upregulated Kac sites [100.0%]), urea cycle (3 Kac sites on 3 Kac proteins and 2 upregulated Kac sites [66.7%]), and ketone body metabolism (1 Kac site on 1 Kac protein and 1 upregulated Kac site [100%]). Most AF/SR differentially expressed Kac sites (148 of 170 Kac sites, 87.1%) on energy metabolism-related Kac proteins were upregulated.

**FIGURE 8:**
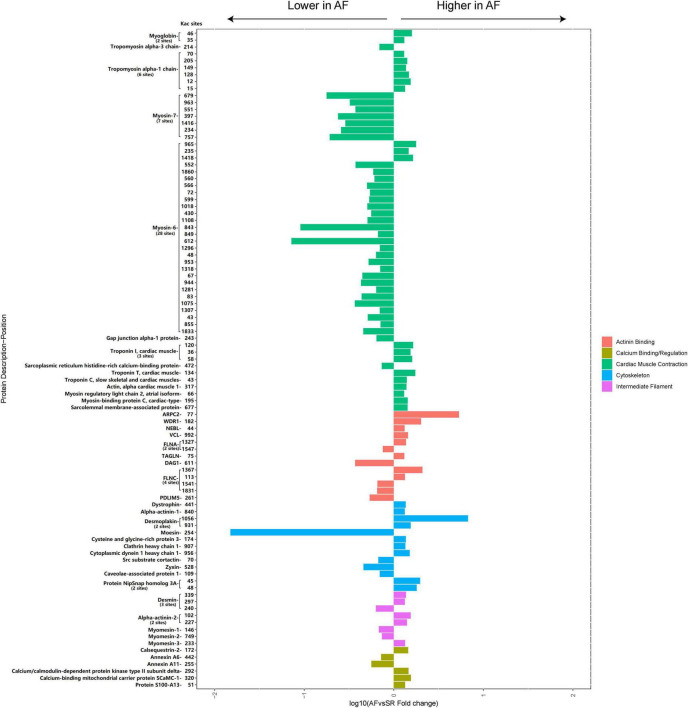
Histogram bar and comparison of the AF/SR differentially expressed cellular contraction- and structure-related acetylated proteins and sites in LAA tissues. AF, atrial fibrillation; SR, sinus rhythm; LAA, left atrial appendage.

A total of 45 cellular contraction and structure-related Kac proteins (accounting for 23.3% of the total AF/SR differentially expressed Kac proteins) had 95 AF/SR differentially expressed acetylated sites (accounting for 27% of the total AF/SR differentially expressed Kac sites) (Figure 8 and Supplementary material 2). These cellular contraction and structure-related Kac proteins were involved in the processes of cardiac muscle contraction (55 Kac sites on 14 Kac proteins) including myosin (36 Kac sites on 3 Kac proteins and 32 downregulated Kac sites [88.9%]) and non-myosin cardiac muscle contraction proteins (19 Kac sites on 11 Kac proteins and 16 upregulated Kac sites [84.2%]), actinin binding (13 Kac sites on 9 Kac proteins and 8 upregulated Kac sites [61.5%]), cytoskeleton (13 Kac sites on 11 Kac proteins and 9 upregulated Kac sites [69.2%]), intermediate filament (8 Kac sites on 5 Kac proteins and 5 upregulated Kac sites [62.5%]), and calcium binding/regulation (6 Kac sites on 6 Kac proteins and 4 upregulated Kac sites [66.7%]).

### Motif analysis

**FIGURE 9:**
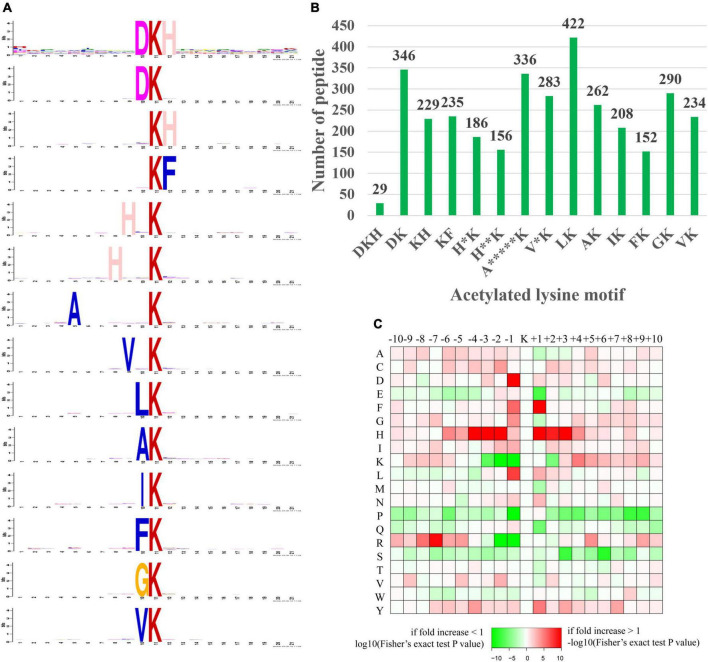
Motif analysis of the identified acetylated peptides. **(A)** Sequence logo of motifs consisting of 20 residues surrounding the targeted lysine residue. **(B)** Number of identified peptides containing each motif. **(C)** Heat map of amino acid frequencies in specific positions, including enrichment (red) or depletion (green) of amino acids flanking Kac sites.

We explored our data set for Kac site-specific sequence motifs by analyzing 20 residues flanking the modified site for overrepresentation and depletion of specific amino acids, revealing general preferences for specific amino acid residues in particular positions surrounding acetylated lysines (Figure 9).

In our study, a total of 14 significantly enriched amino acid sequence motifs from –10 to + 10 surrounding the acetylated lysine were defined, namely, DKacH, DKac, KacH, KacF, H*Kac, H^**^Kac, A^*****^Kac, V*Kac, LKac, AKac, IKac, FKac, GKac, and VKac (* represented a random amino acid residue, Figure 9A). These motifs exhibited different abundances, with the LKac, DKac, A^*****^Kac, GKac, V*Kac and AKac motifs being the most frequent and accounting for 12.37, 10.14, 9.85, 8.5, 8.29, and 7.68%, respectively, of all the identified peptides (Figure 9B).

The amino acid frequencies of the sequences flanking Kac sites were assessed to confirm whether there were position-specific amino acids adjacent to Kac sites with a motif model (Figure 9C). We found that histidine acid (H) was overrepresented in multiple positions (+1, ±2, ±3, and –4) surrounding Kac sites. A preference was observed for aspartic acid (D) and leucine acid (L) in the –1 position, for phenylalanine acid (F) in the +1 position, and arginine acid (R) preferred to appear in the relatively distant –7 position of Kac sites. In addition, lysine acid (K) and arginine acid (R) were both frequently depleted in the –1, –2 position of Kac sites. Serine (S) and glutamic acid (E) were frequently depleted in the +3, +6, and +1 positions of Kac sites. Proline acid (P) preferred to be depleted in multiple positions (+1, +8, and +9) of Kac sites. Interestingly, the occurrence frequencies of histidine acid (H), isoleucine acid (I), and tyrosine acid (Y) surrounding Kac sites were relatively high, while proline acid (P) and serine (S) surrounding Kac sites were usually low.

### Validation results

**FIGURE 10:**
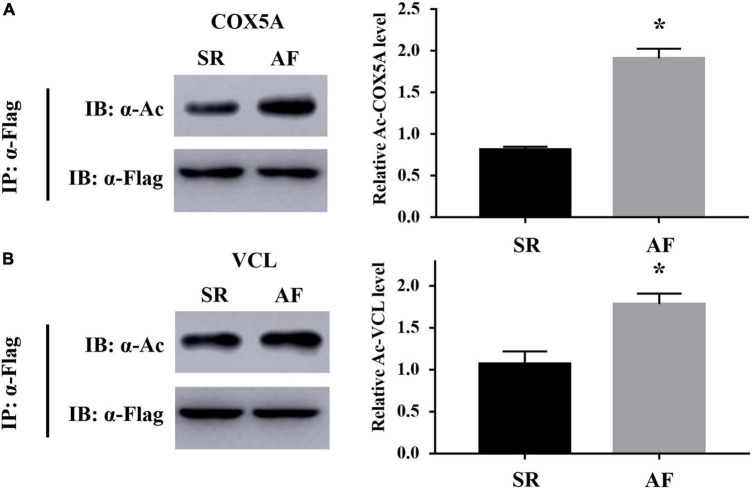
Validation results of acetylated COX5A and acetylated VCL by co-immunoprecipitation (Co-IP) combined with Western blotting (WB). **(A)** Acetylated COX5A. **(B)** AcetylatedVCL. IP, immunoprecipitation; IB, immunoblotting; COX5A, cytochrome c oxidase subunit 5A, mitochondrial; VCL, vinculin. *P < 0.05.

Acetylated cytochrome c oxidase subunit 5A, mitochondrial (COX5A) in energy metabolism-related proteins, and acetylated vinculin (VCL) in cellular contraction and structure-related proteins were validated by IP combined with Western blotting (WB). The results showed that the expression levels of acetylated COX5A (Figure 10A) and acetylated VCL (Figure 10B) were both higher in the LAA tissues from the AF group than those from the SR group (both *P* < 0.05). The results of the validation were consistent with the results of the MS analysis demonstrating the reliability of the results in this study (Figure 2B).

## Discussion

To the best of our knowledge, this is the first study to report the global acetylated proteome of human atrial tissues (LAA tissues), particularly in patients with chronic AF. Compared with published studies (23–25), this study identified the most sites with lysine acetylation modification (5,007 acetylated sites on 1,330 acetylated proteins) in eukaryotic homogenous tissues/cell lysine acetylation modification proteomics. The acetylated levels of 352 acetylated sites on 193 acetylated proteins were differentially expressed between the AF group and the SR group. A total of 231 upregulated acetylated sites on 130 acetylated proteins and 121 downregulated acetylated sites on 74 acetylated proteins were detected in the AF group. The results of the bioinformatic analysis showed that the differentially expressed acetylated proteins were involved in a number of different biological processes and pathways. Among the differentially expressed acetylated proteins, 87 energy metabolism-related Kac proteins had 170 differentially expressed Kac sites, and 45 cellular contraction and structure-related Kac proteins had 95 differentially expressed Kac sites. The results of this study indicated that the differential expression of acetylated proteins in the LAA tissues was involved in initiation and maintenance of AF.

The dynamics of acetylation of protein lysine residuals are tightly controlled by lysine acetyltransferases, transferring the acetyl group of acetyl-CoA to the ε-amino group of lysine residues, and lysine deacetylases, comprising Zn^2+^-dependent histone deacetylases (HDAC1-11) and NAD^+^-dependent silent information regulator 2 family (SIRT1-7) (26, 27). Modulation of acetylation levels by inhibiting HDAC has been shown to attenuate atrial remodeling (28–30). However, the underlying mechanisms remained to be further investigated. Our study provided a landscape of acetylated proteins in patients with AF, and comprehensive analysis of the differential expression profiles of acetylated proteins related to the energy metabolism process, cell structure and function, etc., could facilitate the finding of therapeutic targets.

Growing evidence has shown that posttranslational acetylation of proteins may play a critical role in cardiomyocyte metabolism by modification of the expression and activity of key enzymes in fatty acid β-oxidation, glucose metabolism, and tricarboxylic acid (TCA) cycle (31–33). In rodent animals and humans, a positive correlation has been demonstrated between fatty acid β-oxidation rates and increased acetylation levels of key enzymes controlling oxidation in cardiomyocytes, including long-chain acyl CoA dehydrogenase (LCAD) and L-3-hydroxy acyl-CoA dehydrogenase (β-HAD) (34, 35). Similarly, the significant increased mitochondrial lysine acetylation accompanied by enhanced activities of medium-chain acyl-CoA dehydrogenases (MCAD) and LCAD and higher fatty acid ß-oxidation rates was observed in the heart of diabetic animals (36).

Among proteins involved in fatty acid metabolism, our study identified 36 differentially expressed Kac sites on 16 Kac proteins in the patients with AF, of which 32 Kac sites were significantly upregulated and accounted for 88.9%. There were 25 differentially expressed acetylation sites on 8 Kac proteins related to fatty acid β-oxidation. More prominently, 24 Kac sites on 7 Kac proteins, including MCAD, trifunctional enzyme subunit alpha, 3-ketoacyl-CoA thiolase, carnitine O-acetyltransferase, very long-chain specific acyl-CoA dehydrogenase, and delta (3,5)-delta (2,4)-dienoyl-CoA isomerase, 2,4-dienoyl-CoA reductase, were significantly upregulated in the patients with AF when compared with the patients with SR (Figure 7). Given that acetylation was found to enhance activities of fatty acid β-oxidation in the heart (36), our results suggest that the increased fatty acid oxidation in atrial tissue during AF is closely related to the upregulated acetylation of fatty acid β-oxidation enzymes.

However, the effects of acetylation modification on the activity of metabolic enzymes are complicated depending on metabolic processes and organs. Fukushima et al. showed a significant decline in glycolysis rates concomitant with hyperacetylation of glycolysis enzymes in newborn rabbit hearts, such as hexokinase and phosphoglycerate mutase (35). Meanwhile, inactivation of sirtuin 3 (SIRT3), a member of the silent information regulator 2 family, in myoblasts *in vitro* or in skeletal muscle *in vivo* led to upregulated acetylated level of the pyruvate dehydrogenase (PDH) E1α subunit in K336, an enzyme for carbohydrate metabolism, resulting in decreased PDH enzymatic activity (27). Besides, acetylation of isocitrate dehydrogenase and succinate dehydrogenase, key enzymes in TCA cycle, decreased their activity in cardiomyocytes (37, 38). Also, acetylated proteins of respiratory chain in mitochondria displayed decreased activities, resulting in less production of ATP in various pathological mouse models (15, 39). Furthermore, acetylation of succinyl-CoA: 3-ketoacid-CoA transferase (SCOT) and acetyl-CoA synthetase 2, key enzymes of ketone oxidation, results in decreased enzyme activity, which could be reversed by SIRT3 activation (40–42).

In the present study, most Kac sites of carbohydrate metabolism key enzymes like hexokinase-1 (K488) and PDH (k379, K321) and key enzyme of ketone oxidation SCOT (K511) were hyperacetylated in the LAA tissues of the AF group (Figure 7). Moreover, the vast majority of Kac sites (92.3%) of differentially acetylated metabolic enzymes in TCA cycle, oxidative phosphorylation, and respiratory electron transport chain were upregulated. Many of the upregulated metabolic enzymes are key enzymes in energy production, such as cytochrome c oxidase subunit 5A and ATP synthase subunit d in oxidative phosphorylation, malate dehydrogenase and isocitrate dehydrogenase (NADP) in TCA cycle, and isovaleryl-CoA dehydrogenase in respiratory electron transport chain. Thus, carbohydrate and ketone body metabolism, the main energy source that acts quickly in physiological conditions, is inhibited by modification of acetylation in cardiomyocytes during AF. Meanwhile, the key energy production processes in mitochondria, including TCA cycle, oxidative phosphorylation, and respiratory electron transport chain, also displayed decreased activities under hyperacetylation modification in the heart of patients with AF. Consistently, previous studies have uncovered that the persistently high energy demand ultimately leads to impaired activity of complexes I and II and mitochondrial ATP synthesis and insufficient energy supply in the atrium during AF (7). Reduced glucose uptake during AF in cardiomyocytes might exacerbate the energy insufficiency (43). These findings indicated that the imbalance of energy supply and demand, which is caused by acetylation modification, is consistent with previous findings that unraveled the metabolic remodeling of AF, including impaired carbohydrate metabolism, increased fatty acid β-oxidation, and repressive mitochondrial energy production.

Long-term irregular contractions could induce atrial systolic dysfunction during AF, affecting the progression of AF. Deacetylation by the HDAC family significantly modulates myocardial contractile proteins, repressing the contractility in various pathological models (18, 19, 44, 45). Interestingly, our study found that 32 Kac sites (88.9% Kac sites of myosin) in myosin-7 and myosin-6 were downregulated in the left atrium of the patients with AF, which may contribute to the impaired atrial systolic function during AF.

For cellular structure-related proteins, reduced acetylated alpha-tubulin level in HL-1 cardiomyocytes and canine atrial myocytes may result in myocardial systolic dysfunction (46), and reduced acetylation level of cortactin, an actinin-binding scaffold protein, may partially contribute to development of AF (47, 48). At the same time, increased acetylation level of alpha-tubulin by HDAC6 inhibition could improve myocardial contractility and cardiac function (49). However, the present study showed that 26 Kac sites (65% Kac sites) on 22 cellular structure proteins were upregulated, like vinculin, desmoplakin, and alpha-actinin-2 in the LAA tissues of the AF group. Another 14 downregulated Kac sites related to dystroglycan, moesin, and myomesin-1 were found in the patients with AF. The effects of different acetylated structure proteins on cardiac systolic function are temporarily lacking in relevant studies, and further studies are needed to investigate their role in regulation of cardiac function.

Based on the profound effects of acetylation on energy metabolism and cardiac contractile proteins, we posit that modulation of acetylation levels in metabolic enzymes and contractile proteins might present a new therapeutic target for AF treatment.

### Limitations

Several limitations of the current study should be considered. First, despite the fact that a comprehensive analysis was conducted, stringent database search methods were used to minimize the FDR (<1%), and the set minimum score for modified peptides was >40, the sample size of LAA tissues in this study is relatively small and there remains the possibility of limited statistical power or residual confounding that could cause bias. Second, the cell types (such as atrial myocytes and fibroblasts) in LAA tissues in this study were not distinguished, so further studies are needed to verify the differential expression of acetylated proteins at the level of atrial myocytes during AF. Third, we did not perform an acetylated proteomic study on LAA tissues from patients with paroxysmal AF in this study.

## Conclusion

In summary, this study represented the first report and the most comprehensive description of acetylated proteomics on patients with valvular heart disease with AF. Many differentially expressed acetylated proteins related to numerous processes and pathways like energy metabolism and cellular contraction were found in LAA tissues between patients with chronic AF and those with SR, which may reflect the impaired ATP production capacity and decreased atrial muscle contractility in the atrium during AF. Thus, we suggest that acetylation may play an important regulatory role in metabolic and contractile remodeling of the atrium during AF. The identified numerous new acetylated sites and proteins in LAA tissues of patients with AF may become promising targets for prevention and treatment of AF.

## Data availability statement

The raw data supporting the conclusion of this article will be made available by the authors, without undue reservation.

## Ethics statement

The studies involving human participants were reviewed and approved by the Ethics Committee of the Second Xiangya Hospital of Central South University. The patients/participants provided their written informed consent to participate in this study.

## Author contributions

TT contributed to design, data curation, and writing (original draft preparation). QL and FQ contributed to conceptualization, design, and writing. FB contributed to methodology and investigation. YX contributed to supervision. CS and XL contributed to specimen collection and processing. BL and NL contributed to validation. YM and BZ contributed to reviewing and editing. SZ contributed to design and supervision. All authors contributed to the article and approved the submitted version.

## Footnotes

1
http://www.ebi.ac.uk/GOA/

2
http://www.ebi.ac.uk/interpro/

3
http://www.genscript.com/psort/wolfpsort.html

4
http://www.genome.jp/kegg/

5
http://www.genome.jp/kaas-bin/kaas_main

6
http://www.kegg.jp/kegg/mapper.html

7
http://mips.helmholtz-muenchen.de/genre/proj/corum/index.html

8
https://metacpan.org/pod/Text::NSP::Measures::2D::Fisher

9
https://cran.r-project.org/web/packages/cluster/

10
https://cran.r-project.org/web/packages/networkD3/

11
http://meme-suite.org/tools/momo
