# Supplementary material for: Quantitative acetylated proteomics on left atrial appendage tissues revealed atrial energy metabolism and contraction status in patients with valvular heart disease with atrial fibrillation
Source: Front Cardiovasc Med. 2022 Sep 13;9:962036. doi: 10.3389/fcvm.2022.962036 (PMC9513032; doi:10.3389/fcvm.2022.962036)
Supplement: Supplementary file 3 [file Data_Sheet_1.PDF]

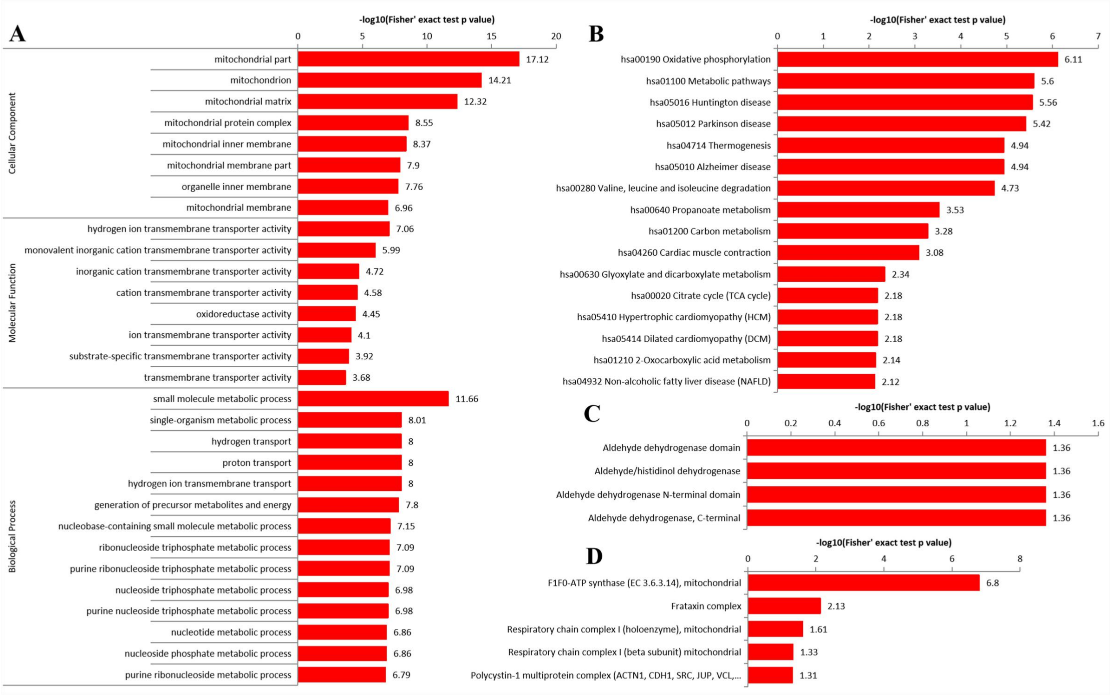


**Supplementary Figure 1. Enrichment analysis of GO, KEGG pathway, protein domain and protein complex of the up-regulated acetylated proteome in LAA tissues.**

**A,** GO (molecular function, cellular component and biological process). **B,** KEGG pathway. **C,** Protein domain. **D,** Protein complex. GO, Gene Ontology; KEGG, Kyoto Encyclopedia of Genes and Genomes; AF, atrial fibrillation; SR, sinus rhythm; LAA, left atrial appendage.

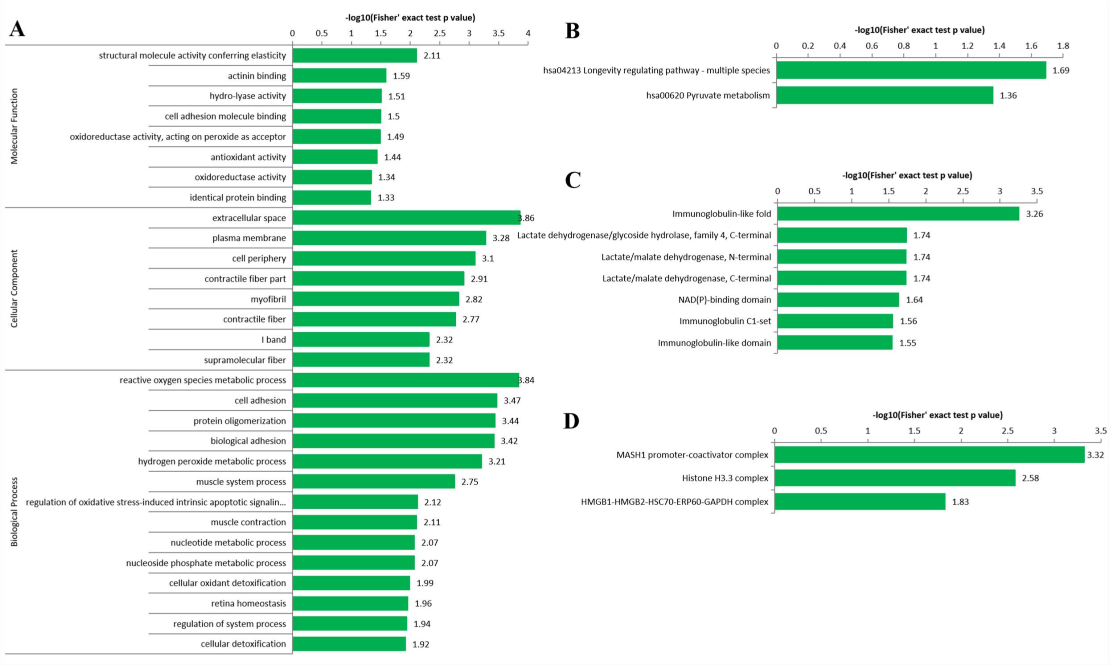


**Supplementary Figure 2. Enrichment analysis of GO, KEGG pathway, protein domain and protein complex of down-regulated acetylated proteome in LAA tissues.**

**A,** GO (molecular function, cellular component and biological process). **B,** KEGG pathway. **C,** Protein domain. **D,** Protein complex. GO, Gene Ontology; KEGG, Kyoto Encyclopedia of Genes and Genomes; AF, atrial fibrillation; SR, sinus rhythm; LAA, left atrial appendage.
